# Restorative Rehabilitation of a Patient With Tooth Wear: A One-Year Clinical Follow-Up Report

**DOI:** 10.7759/cureus.37798

**Published:** 2023-04-18

**Authors:** Vimal Kumar, Satheesh Reddy, Santoshi Kumari V, Riyaz Basha, Nirban Mitra

**Affiliations:** 1 Prosthodontics, Mallareddy Institute of Dental Sciences, Hyderabad, IND; 2 Prosthodontics, Malla Reddy Institute of Dental Sciences, Hyderabad, IND

**Keywords:** ace classification, erosive tooth wear, full-mouth rehabilitation, minimal intervention, palatal veneers

## Abstract

Tooth wear is a multifactorial process of complex aetiology. It may be considered a physiological or pathological process depending upon the rate and degree of occurrence. The patients may present with symptoms of sensitivity, pain, headaches or recurrent loss of restorations and prostheses, leading to loss of function. This case report describes the rehabilitation of a 65-year-old male patient with intrinsic dental erosion combined with generalised attrition. The restorative treatment aimed at restoring anterior guidance, establishing a stable occlusion for the patient with minimal intervention.

## Introduction

Tooth wear or tooth surface loss is defined as tooth tissue loss due to attrition, abrasion, and erosion. It is an insidious disease with a complex aetiology and may be considered multifactorial [1,2]. This is commonly considered to be a physiological process occurring due to ageing. However, the rate and degree of tooth wear would indicate whether the process is physiological or pathological [1]. Despite the technological advancements in the field of diagnosis and treatment, leading to improved oral health conditions, there is a noticeable increase in the prevalence of tooth wear, which is reported to be 77% among the dentate population [3]. Several global studies have concluded that changes in lifestyle and dietary habits are among the potential contributing factors leading to a higher prevalence of tooth wear [3,4].

Tooth wear resulting in the loss of enamel and dentin may be asymptomatic in patients with physiological tooth wear or may have a negative effect on teeth with pathological wear, thus influencing the individual’s quality of life. The signs and symptoms observed depend upon the cause of the disease. However, the common features include sensitivity, pain, headaches, recurrent dislodgement of restorations or prostheses and loss of anterior and posterior teeth, resulting in occlusal disharmony [5].

The restoration of worn teeth requires a holistic approach with an accurate diagnosis, an analysis of the degree of structural damage, and the drafting of a precise treatment plan with minimal intervention. The improved understanding of the pathophysiology of tooth wear has led to the establishment of biologically driven treatment strategies for management that involve advanced biomaterials and newer techniques with minimal intervention [6].

This case report discusses the comprehensive rehabilitation of a patient diagnosed with tooth wear, including dental erosion and generalised attrition. The treatment aimed at restoring the anterior guidance, improving the aesthetics and establishing a stable occlusion for the patient with minimal intervention.

## Case presentation

A 65-year-old male patient reported to the Department of Prosthodontics and Crown & Bridge with worn-out dentition. He complained of broken lower front teeth and a broken fixed denture and desired replacement. He also reported frequent dislodgement and chipping of the same fixed denture fabricated five years ago by a general dentist. He also complained of generalised sensitivity, thinning of the upper front teeth, and food lodgement in the lower left back tooth region.

**Figure 1:**
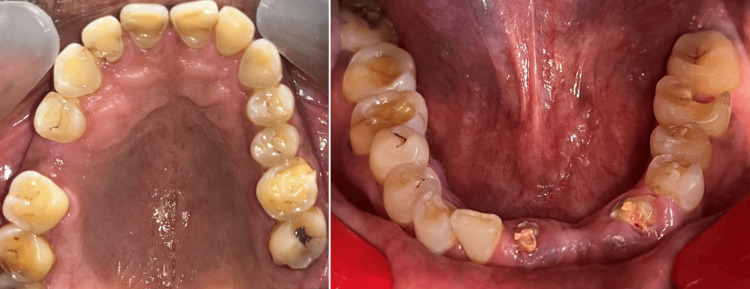
Preoperative view of the maxillary and mandibular arch

The patient presented with a medical history of gastroesophageal reflux disease and was on medication for the condition. The examination of the temporomandibular joint presented with no clicking or deviation. The intraoral clinical examination revealed a missing right upper first molar and left lower central and lateral incisors and a dislodged fixed partial denture with extensively damaged lower front teeth (Figure 1).

The clinical examination of teeth from right to left maxillary canines, revealed extensive erosive damage on the palatal aspect with exposed dentin and short nonaesthetic crowns. The tooth wear lesions exhibited an intact enamel surface along the palatal gingival margin. They were classified as Class III based on the anterior clinical erosive (ACE) classification [7]. The maxillary and mandibular posterior teeth presented a flattened cusp morphology, glossy occlusal surfaces and abrasive defects. There was also a lack of anterior guidance and signs of functional occlusal interferences.

Full arch diagnostic impressions for the fabrication of diagnostic casts were taken for the maxilla and mandible using elastomeric putty impression material (Aquasil Soft Putty; Dentsply Sirona, USA). A diagnosis of the tooth surface lesions, including in terms of erosion and attrition, was established for the patient. This was based on a clinical radiographic examination augmented by a diagnostic evaluation.

The extensively damaged left mandibular canine and right central incisor required root canal therapy and a cast post to retain the cores. A cast post was planned due to an oval root canal teeth anatomy. The left mandibular first molar tooth presented a fractured distobuccal cusp, with normal lamina dura and no pulpal involvement.

**Figure 2:**
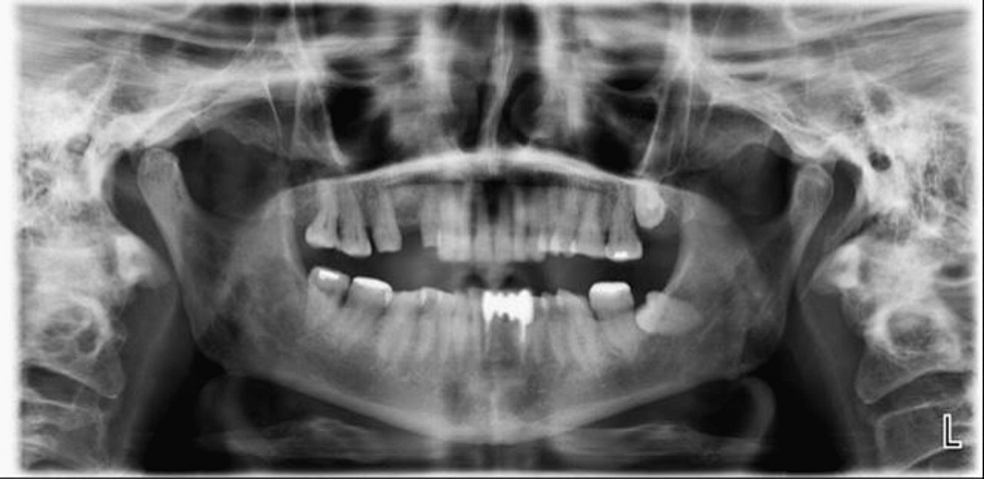
OPG image after completion of post and core Orthopantomography (OPG) image showing the preoperative status of tooth wear.

**Figure 3:**
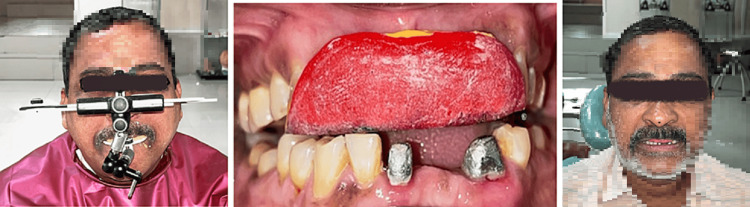
Interocclusal records obtained using facebow

On a subsequent appointment, after the completion of the root canal treatment, post and core, diagnostic casts were mounted on a semi-adjustable articulator (A7 Plus, Bio-Art, Confident Pvt Ltd, India) using facebow (Elite BioArt, Confident Pvt Led, India) orientation, and interocclusal records were obtained and an orthopantomograph was taken (Figure 2). The centric and dynamic occlusal contacts were assessed via diagnostic mounting on the articulator (Figure 3).

Considering the biologically based restorative treatment strategies, a comprehensive functional rehabilitation was planned to restore function, replace the missing teeth and re-establish stable occlusion and aesthetics. Due to economic constraints, the missing teeth were replaced by a metal-ceramic fixed partial denture. The ACE Type III palatal erosive lesions were rehabilitated with palatal indirect composite veneers. The other teeth with erosion and attrition were rehabilitated using bonded restorations.

**Figure 4:**
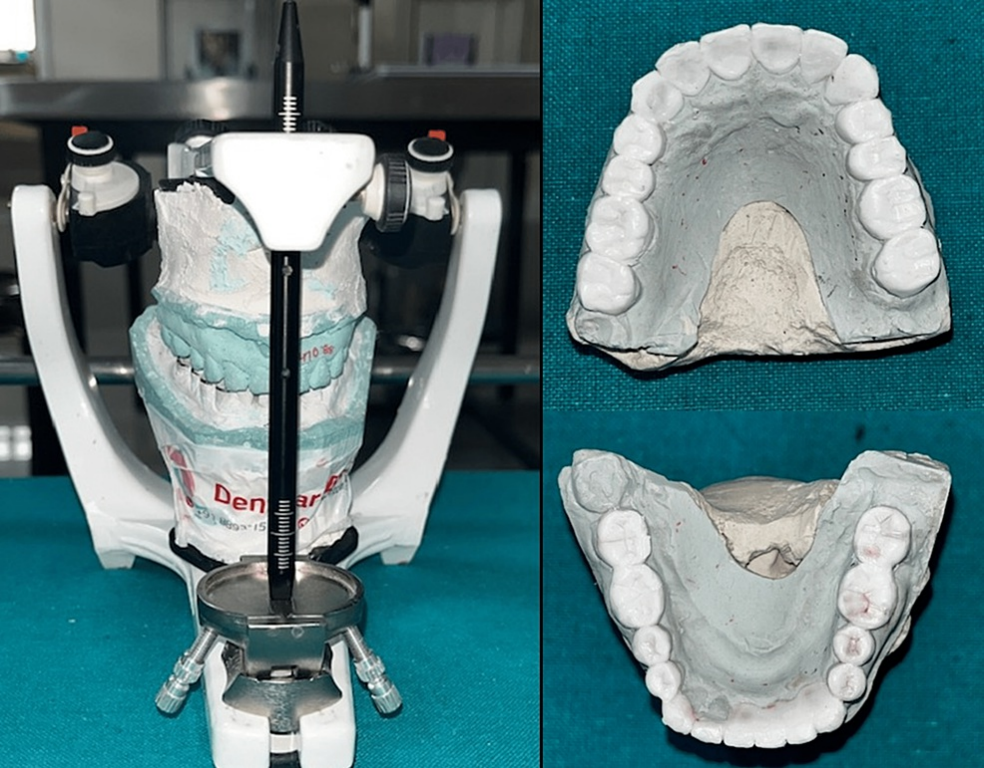
Anterior guidance established by the anterior prosthesis

On a subsequent appointment, the vertical dimension was re-established by increasing the vertical dimension by 1.5 mm. The occlusal plane was established using a Broadrick occlusal plane analyser (Bio-Art, Confident Pvt Ltd, India). The condylar guidance was recorded using centric and eccentric interocclusal records. A diagnostic wax-up was performed to establish accurate anterior guidance and to verify the aesthetic and functional modification of the dentition. This relation was transferred to the patient’s mouth through interim restorations (Luxatemp, Hamburg, Germany) fabricated using a silicone putty index (Aquasil Soft Putty, Dentsply Sirona, USA). The interim restorations were placed for three weeks (Figure 4).

**Figure 5:**
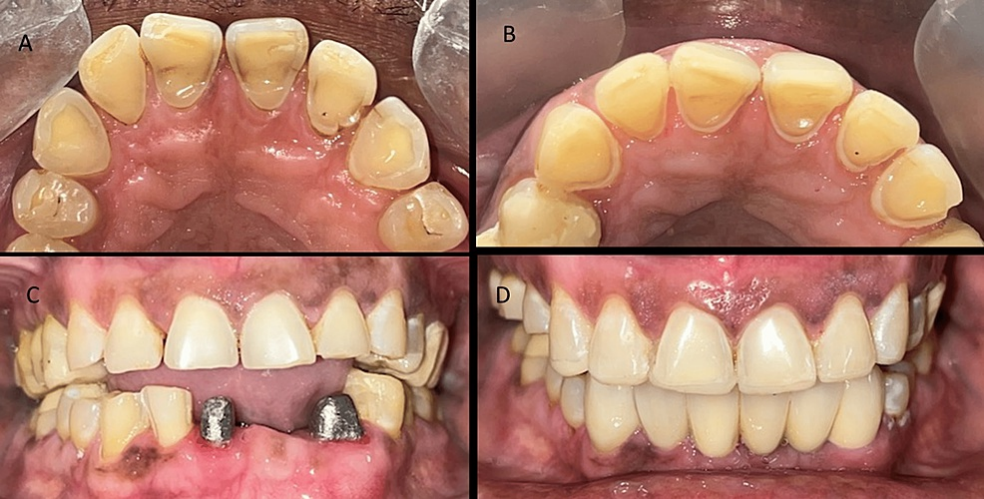
Postoperative view with the maxillary and mandibular prosthesis

At the end of the three weeks, the patient was evaluated for any discomfort or fracture of the interim restorations. Following this, the maxillary and mandibular anterior teeth were replaced with permanent prostheses. Since the maxillary anterior exhibited an ACE Type III erosive wear pattern, indirect composite palatal veneers were fabricated after minimal tooth preparation. The lower missing teeth were replaced by a porcelain-fused-to-metal fixed partial denture in the right mandibular lateral incisor and the right mandibular canine and restored using lithium disilicate veneers (Figure 5).

A porcelain-fused-to-metal fixed partial denture was placed for the following maxillary teeth. The right first molar with the right maxillary second premolar and right second molar as abutments. The right maxillary first and second premolar, the right mandibular first and second premolar and the left maxillary first molar were rehabilitated using lithium disilicate veneer overlays. This was followed by rehabilitating the left and right mandibular first molar with an overlay and a tabletop prosthesis, respectively.

**Figure 6:**
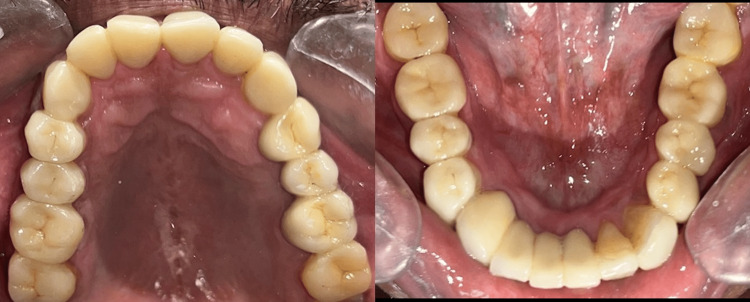
Postoperative View with the Maxillary and Mandibular Prosthesis

Rehabilitation of the maxillary left and right second molar, and the right mandibular second molar was performed at the end of the treatment using lithium disilicate tabletop prosthesis. The final fixed partial dentures and bonded restorations were cemented using the standard veneer cementation protocol (Variolink Veneer, Ivoclar Vivadent) (Figure 6).

**Figure 7:**
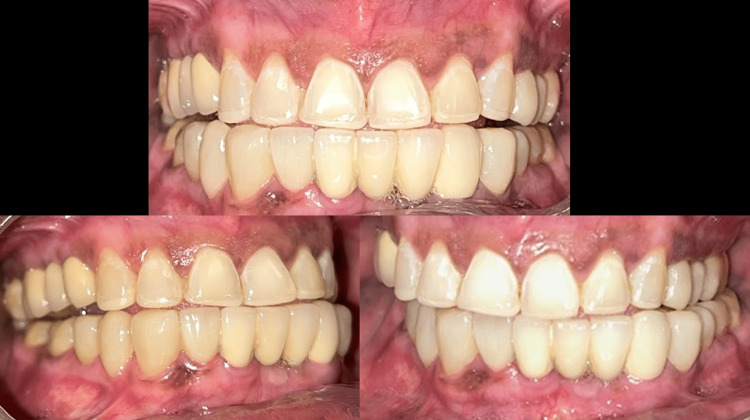
Clinical follow-up after six-months

Occlusal adjustments were performed over two follow-up appointments with a one-week interval. The patient was counselled on oral hygiene practices and the dietary and lifestyle modifications necessary for maintaining the prosthesis. The patient was evaluated postoperatively every six months (Figure 7).

**Figure 8:**
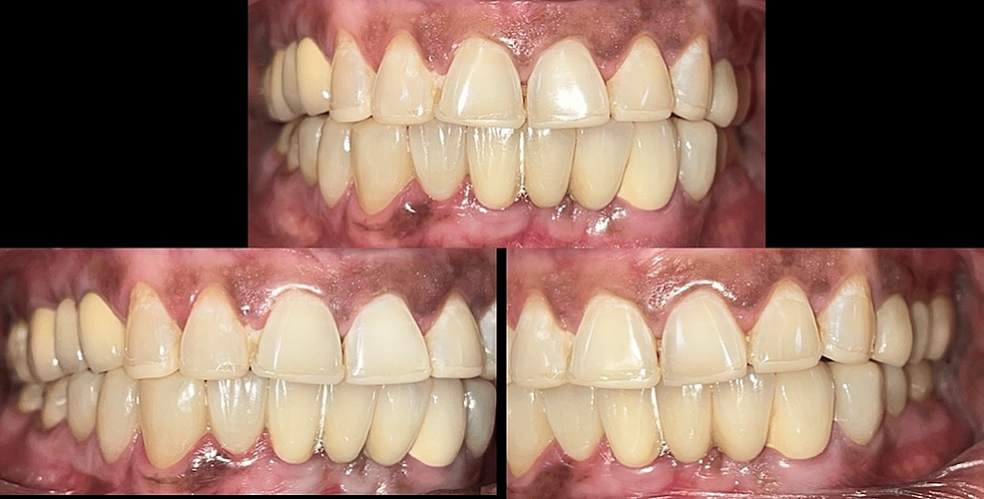
One-year clinical follow-up

**Figure 9:**
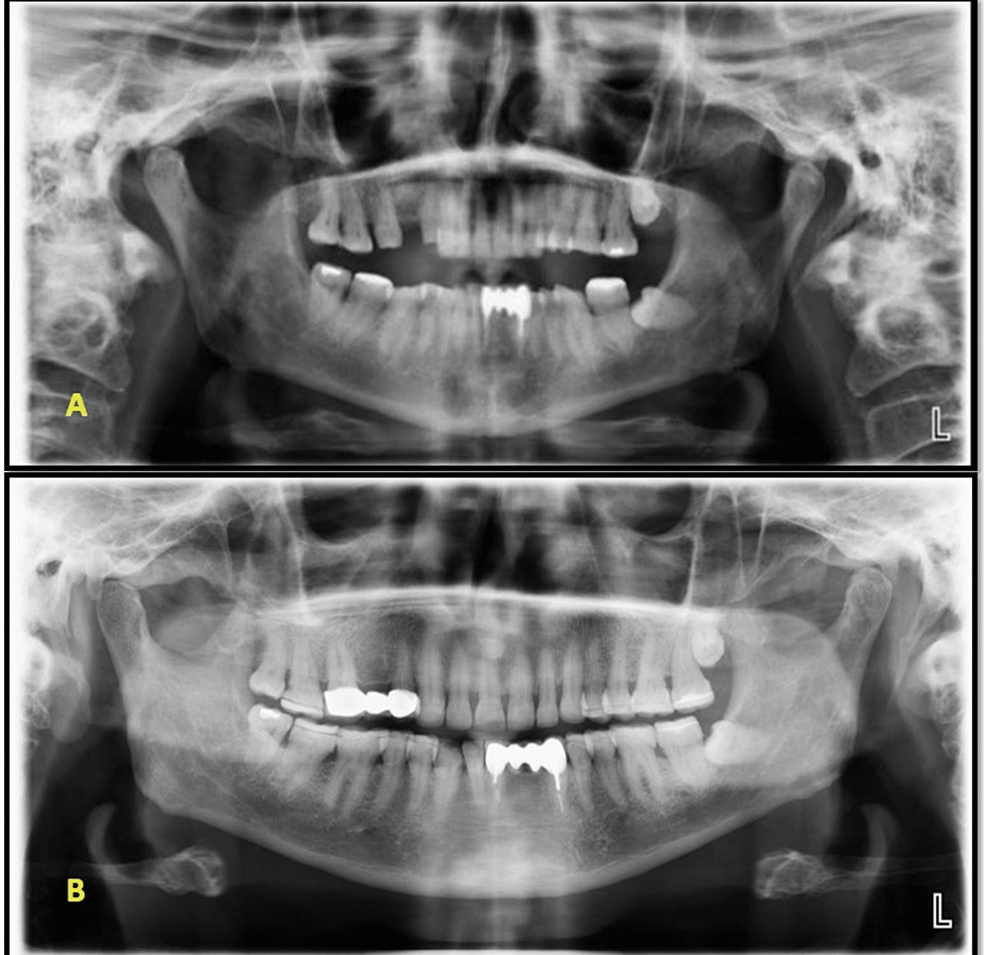
Preoperative and postoperative radiographs A: preoperative radiograph, B: one-year postoperative radiograph

A one-year clinical and radiographic examination revealed good prosthesis adaptation; the patient was comfortable and content (Figures 8, 9).

## Discussion

Tooth wear is a multifactorial process occurring when various tooth wear mechanisms, such as attrition, abrasion and erosion, act independently or in combination [6]. The first line of management involves the identification of the aetiology and specific measures to prevent further damage. In the present case, the tooth wear was due to erosion and attrition, resulting in disturbed posterior occlusion.

A treatment protocol following the principles of minimal intervention was established that included a thorough investigation of the aetiology, a biologically based treatment to preserve the tooth vitality, maximise the retention of tooth structure and improve the aesthetics.

The first step in the treatment was to establish the lost vertical dimension, the anterior guidance and the plane of occlusion. The extensive tooth wear of the maxillary anterior teeth resulted in teeth thinning with a reduction in incisal height. This adversely affected the horizontal overlap and the anterior guidance, resulting in posterior teeth interferences. Therefore, harmonising the lingual contours of the maxillary anterior teeth with the facial contours of the mandibular incisors was crucial.

In the present case, a combination occlusal scheme with canine-guided occlusion on the right side and group function occlusion on the left side was implemented. This specific occlusal pattern helped us to relieve any posterior-lateral stresses on the non-occluding teeth [8,9].

A diagnostic mock-up with composite temporisation allows the clinician to analyse the response from the muscles of mastication and temporomandibular joint to the newly established occlusion, and to verify the new centric position [10]. It also allows for eliciting the patient’s opinion regarding the tooth’s form, shape and inclination.

The classification by Vailati and Belser provides various treatment options for the patient’s teeth based on the amount and location of tooth surface loss [7]. The present case clinically exhibited distinct dentin exposure for the maxillary anterior teeth, with <2 mm of damage to the incisal edge length. This confirmed a Class III dental erosion. These teeth appeared to have a smooth, shiny, glossy dentin surface with a thin collar of enamel cervically. Therefore, indirect composite veneers were planned since they require minimal tooth preparation and offer a predictable bond on dentin compared to lithium disilicate, with the latter relying on intact enamel for bonding. Furthermore, indirect composite veneers are easily repairable [10,11].

Schnuyler CH described the various treatment strategies based on the amount of loss in the vertical dimension [12]. Restorations with direct composites are recommended for a vertical dimension loss of <2 mm. In comparison, indirect ceramic veneer and overlays are recommended for a vertical dimension loss of >2 mm. Indirect ceramic restorations are preferable for the rehabilitation of erosion with a vertical dimension loss of >4 mm.

Conventional crowns are effective in restoring missing or extensively worn teeth. They are advantageous since the provisional restorations help in the evaluation of the aesthetics and function from the dentist’s and patient’s perspectives [13,14].

Patients with dental erosion must follow certain lifestyle and dietary modifications. They may also require counselling for the same. Postoperative follow-up care is important in terms of evaluating and monitoring the functioning of the prostheses.

## Conclusions

Tooth surface lesions are multifactorial conditions that strongly influence the quality of life of a patient. With the change in lifestyle and dietary habits among the population, there is an increase in the prevalence of these conditions. Therefore, the dentist must accurately diagnose these conditions at the initial stages and incorporate preventive measures to avoid and minimise structural loss of the enamel and dentin. Comprehensive diagnostic criteria used to identify the etiological factors and the extent of tooth structure loss enable the dentist to formulate an appropriate treatment plan and select the correct material for long-term success.

## References

[1] Bartlett D, Dugmore C (2008). Pathological or physiological erosion--is there a relationship to age?. Clin Oral Investig.

[2] Bomfim DI (2010). Quality of Life of Patients with Different Levels of Tooth Wear, M.Sc. thesis, Department Of Prosthodontics, Eastman Dental Institute At The. Quality of Life of Patients with Different Levels of Tooth Wear (M.Sc. thesis).

[3] (2011). Adult Dental Health Survey 2009 - Summary report and thematic series. Survey.

[4] Van't Spijker A, Rodriguez JM, Kreulen CM, Bronkhorst EM, Bartlett DW, Creugers NH (2009). Prevalence of tooth wear in adults. Int J Prosthodont.

[5] Wazani BE, Dodd MN, Milosevic A (2012). The signs and symptoms of tooth wear in a referred group of patients. Br Dent J.

[6] Kelleher MG, Bomfim DI, Austin RS (2011). Biologically based restorative management of tooth wear. Int J Dent.

[7] Vailati F, Belser UC (2010). Classification and treatment of the anterior maxillary dentition affected by dental erosion: the ACE classification. Int J Periodontics Restorative Dent.

[8] Thornton LJ (1990). Anterior guidance: group function/canine guidance. A literature review. J Prosthet Dent.

[9] Sidana V, Pasricha N, Makkar M, Bhasin S (2012). Group function occlusion. Indian J Oral Sci.

[10] Darbar UR, Hemmings KW (1997). Treatment of localized anterior toothwear with composite restorations at an increased occlusal vertical dimension. Dent Update.

[11] Mizrahi B (2004). A technique for simple and aesthetic treatment of anterior toothwear. Dent Update.

[12] Schuyler CH (2001). The function and importance of incisal guidance in oral rehabilitation. 1963. J Prosthet Dent.

[13] Williamson EH, Lundquist DO (1983). Anterior guidance: its effect on electromyographic activity of the temporal and masseter muscles. J Prosthet Dent.

[14] Morris JB (2008). Functional occlusion: from TMJ to smile design. Journal of Prosthodontics.

